# A Modified Method for DNA Extraction From Buccal Cells for Genetic Epidemiological Studies Among Children

**DOI:** 10.7759/cureus.76140

**Published:** 2024-12-21

**Authors:** Wendy Yesenia Escobar de González, Katleen Argentina Aguirre de Rodríguez, Vianney Castañeda Monroy, Nuria Patiño-Marín, Carlo E Medina-Solís, Nereyda Niño-Martínez, Yolanda Terán-Figueroa

**Affiliations:** 1 Research Center, Faculty of Dentistry, University of El Salvador, San Salvador, SLV; 2 Health Research and Development Center, University of El Salvador, San Salvador, SLV; 3 Department of Clinical Research, University of San Luis Potosí, San Luis Potosí, MEX; 4 Advanced Studies and Research Center in Dentistry "Dr. Keisaburo Miyata" School of Dentistry, Autonomous University of State of Mexico, Toluca, MEX; 5 Academic Area of Dentistry of Health Sciences Institute, Autonomous University of Hidalgo State, Pachuca, MEX

**Keywords:** dna, epidemiology, genetics, molecular biology, pcr test

## Abstract

Background: In large-scale molecular studies, a protocol that generates high yields and quality DNA for future polymerase chain reaction (PCR) assays is needed. The collection of buccal cells by cytobrush may represent an efficient, noninvasive, and inexpensive method for obtaining genetic material from school populations. The aim of this study was to develop a method to obtain genomic DNA from buccal cells of schoolchildren, and the DNA was extracted immediately after collecting the buccal cell samples and after storing the samples for 8 months at -20 °C to establish the feasibility of the method for epidemiological studies.

Methods: Forty-five Salvadoran schoolchildren aged between seven and fifteen years participated. Two samples of buccal cells were collected with cytobrush from each subject. The yield of the extracted DNA was evaluated by spectrophotometry, and purity was measured using optical density (OD) 260/280. The functional quality was assessed by PCR and established by amplifying the 536 bp region of the *β-hCG* gene.

Results: The yield was 65.70 μg for the immediate extraction group and 47.63 μg for the extraction group after eight months of storage at -20°C. The purity measured was 2.0 for immediate extraction and 1.9 for extraction after eight months. The functional quality was greater than 90% for both groups.

Conclusion: This protocol can be used to obtain DNA of high yield, purity, and functional quality from schoolchildren for genetic epidemiological studies based on PCR. This method is effective whether DNA is extracted immediately after collection or after buccal cell samples have been stored at 20°C for eight months.

## Introduction

In the era of genomics, research in molecular epidemiology has substantially contributed to knowledge of the pathogenesis of human diseases. It provides major benefits to health care through the potential design of individualized therapies based on genetic profiles [1,2]. For studies in this field, it is essential to obtain genomic DNA of sufficient quantity and quality, both for immediate analysis and for future use in different assays [3,4]. Methods of collection should be simple, inexpensive, and noninvasive, which is essential for improving the participation rates of study subjects, especially in the case of multicenter studies [5-7].

For a long time, the preferred source of human genetic material has been peripheral blood. However, collecting peripheral blood samples is invasive and expensive and can lead to limitations in donor collaboration, particularly in pediatric populations that also need a phlebotomist. [8,9]. For these reasons, DNA extracted from buccal cells is gaining recognition as an efficient, painless, low-risk, and inexpensive alternative source for large-scale clinical trials and research [10,11]. In addition, no specific training is required to collect buccal cells. The most commonly used methods for the collection of buccal cells are rinses and swabs or cytobrushes [3,12,13]. Among these, the cytobrush protocol offers many advantages, such as easy sample collection and convenience for the patient [10,14]. In addition, Polymerase Chain Reaction (PCR) has been used in several studies to obtain materials suitable for DNA amplification [7,15-17].

Regarding DNA extraction from samples, different kits are available on the market, but most of them are produced in industrialized countries, and their accessibility at affordable prices in developing countries is limited. Consequently, several protocols have been developed to extract DNA from buccal cells; however, there is no clear agreement on several aspects, such as the effect of storing the samples for an ideal period of time [18,19]. In addition, few trials have involved samples from school-age populations [13]. Therefore, the aim of this study was to obtain genomic DNA from the buccal cells of Salvadorian schoolchildren and to compare the yield and quality of the DNA extracted immediately and after storage for eight months at -20°C to establish the viability of the method for epidemiological studies. Some modifications were made in relation to protocols used in other investigations to establish the viability of the method proposed here for epidemiological studies.

## Materials and methods

Study population

Salvadoran schoolchildren with no relevant medical history aged 7-15 years were included in the study. Subjects who had taken any medication on the day of collection were excluded. Sampling was by convenience; forty-five schoolchildren participated. This study was approved under protocol number CEI-FOUES-2022-012 by the Ethics Committee of the Faculty of Dentistry of the University of El Salvador, located in San Salvador, El Salvador. The principles of the Declaration of Helsinki were complied with, and written informed consent was obtained from the legal guardians and the assent of all subjects.

Collection of buccal cell samples

In our protocol, to prevent contamination due to food residues, the examiner cleaned each schoolchild's mouth with a new toothbrush and pumice stone. The subjects were instructed to rinse twice with 30 mL of purified water.

To collect the buccal epithelial cell samples, we performed the method described by Mullot et al. [11]. A sterile cytobrush (Medstar, China) was rotated on the inner sides of both cheeks of the subject for 15 seconds, three times on each side. The cytobrush was suspended in a 2 mL microcentrifuge tube (Eppendorf, USA) containing 1 mL of lysis buffer (10 mM Tris-HCl, pH 7.8; 5 mM EDTA; 0.5% SDS), and the stick was cut with scissors. In contrast to Mullot [11], who kept his samples at room temperature, in our protocol, each tube with its sample was sealed and stored in a polyurethane cooler at a temperature of 2-4°C, in which they were shipped to the laboratory for processing on the same day. One week later, a second sample was collected from each subject following the same protocol. This second group of samples was stored at -20°C in the laboratory for eight months until DNA extraction.

Extraction of the buccal genomic DNA

Genomic DNA was extracted following a protocol modified from that described by Ghatak et al. for samples collected with buccal swabs in an adult population [8]. After thawing, the cytobrushes were removed by rubbing against the walls of the tubes to dislodge the epithelial cells and were subsequently discarded. For each sample suspended in 1 mL of buffer, 5 μL of proteinase K (20 mg/mL) (Promega, USA) was added. These samples were vortexed (Bunsen, Spain) and incubated at 55°C overnight to prolong the function of proteinase K in protein digestion and to eliminate contamination, as performed by Küchler [7]. The next day, 700 μL of chloroform: isoamyl alcohol (24:1) (Sigma Aldrich, USA) was added, and the mixture was vortexed for 5 seconds, as this was identified as sufficient time to separate the nucleic acids. Subsequently, the samples were centrifuged (Thermo Scientific Sorvall ST 8R; UK) at 14,000 g at 4 °C for 20 minutes. The upper aqueous phase was transferred to a new sterile 2 mL microcentrifuge tube with the addition of 462 μL of cold isopropanol ≥99.5% (Sigma Aldrich, USA) and 70 μL of 3 M sodium acetate (Sigma Aldrich, US). In order to enhance the yield, the precipitation time was taken from the Zayats protocol [12]. The solution was mixed by gently inverting the tube 5 times and stored at -20 °C overnight. The sample was then centrifuged at 14,000 g at 4°C for 20 min. The supernatant was decanted, and 1 mL of cold 70% ethanol was added to ensure proper washing in one step. The tube was gently inverted 5 times to wash the pellet, which was subsequently centrifuged at 14,000 g at 4 °C for 10 minutes. The ethanol was decanted, and the tubes were inverted, leaving the DNA pellet to dry at room temperature for 2 hours on DNAase-free absorbent paper. The dried pellet was resuspended in 50 μL of ultrapure endonuclease-free water (Invitrogen, US) and stored at -20 °C. This process was performed for the first buccal cell sample from each subject immediately after collection and for the second sample after eight months of storage at -20°C.

Yield and purity of genomic DNA

DNA yield and purity were assessed spectrophotometrically using a Genova Nano Spectrophotometer (Jenway, UK). The DNA concentration was determined by 260 nm absorbance readings, considering that an absorbance of 1 unit at 260 nm corresponds to 50 µg genomic DNA per mL. The purity was determined from the 260/280 nm ratio, including acceptance criteria for purity between 1.6 and 2.

DNA integrity

The samples were prepared by adding 2 µl of DNA, 2 µl of ultrapure endonuclease-free water (Invitrogen, US), and 1 µl of DNA loading buffer (Thermo Scientific, US) to 0.5 mL microtubes. Genomic DNA integrity was determined on a 1% agarose gel (VWR, USA) with 0.5 µl of GelRed (Biotium, USA) by electrophoresis (Thermo Scientific Owl EC105, US) for 2 hr at 70 V, followed by visualization by ultraviolet transillumination in a gel documenter (UVP GelDoc-It, US) gel documenter, UVP GelDoc-it, US). Sample migration was observed against a 100 base pairs (bp) marker (Maestrogen, England). The genomic DNA was verified to be larger than 23 kilobases (kb).

DNA quality

The functional quality and suitability of the extracted DNA for PCR-based epidemiological studies were determined by amplifying a 536 bp amplicon of the human β-globin gene, as reported by Greer et al. [20]. The primers RS42 (5'-GCTCACTCAGTGTGGCAAAG-3') and KM29 (5'-GGTTGGCCAATCTACTCCCAGG-3') were used to generate the expected fragment. The reaction mixture was prepared for a total volume of 25 μL, containing 2 μl DNA (50 ng/μL); 12.5 μL master mix 2X (Promega, US), 1 μM each primer, 1 μM DMSO (Thermo Scientific, US) and 7.5 μL nuclease-free Water (Invitrogen, US). Conventional PCR was performed in the thermal cycler (Mini Amp, Applied Biosystems, US), subjecting the samples to 39 amplification cycles at 95°C for 1 minute, 55°C for 1 minute, and 72°C for 2 minutes, followed by a final extension of 5 minutes at 72 °C. The amplification products were evaluated by 2% agarose gel electrophoresis at 70 V for 2 hours. The migration of PCR products on the gel was observed against a 100 base pair (bp) marker (Maestrogen, England) and evaluated on a gel documenter (UVP GelDoc-It, USA).

Statistical analysis

Analyses were performed using the SPSS statistical package, version 25 (SPSS Inc., Chicago, IL, USA). Normality was assessed using the Shapiro‒Wilk test, and differences in yields and A260/A280 ratios between DNA extraction time points (immediately and after eight months) were analyzed using the Wilcoxon matched-pairs signed-rank test. Differences were considered statistically significant at p<0.05. The data are presented as the means±SD, median, and interquartile range (IR).

## Results

As measured spectrophotometrically at 260 nm, the buccal cell DNA extraction process yielded an average of 65.70 μg for immediate extraction and 47.63 μg for extraction after eight months of storage at -20°C. The mean 260/280 OD ratio was 2.0 for immediate extraction and 1.9 for extraction after eight months (Table 1).

**Table 1:** Comparison of the buccal cell DNA yield (μg) and purity (absorbance ratio 260:280 nm) obtained from samples processed immediately after cytobrush collection and after 8 months of storage at −20°C. SD: standard deviation;  IR: interquartile range

Time of extraction	Yield (μg)		Purity (ratio A260/A280)	
	Mean (SD)	Median (IR)	Mean (SD)	Median (IR)
Immediately	65.70 (74.49)	39.94 (68.37)	2.0 (0.37)	2.1 (0.28)
After 8 months	47.63 (33.47)	39.58 (41.49)	1.9 (0.26)	1.9 (0.30)

According to the results of the statistical analysis for immediate extraction and stored samples, there were no significant differences regarding the yield (with p=0.488) or the purity (with p=0.172). The results were compared with those reported in the literature (Table 2). 

**Table 2:** Comparison of yield and purity of genomic DNA from buccal cells collected by the cytobrush method. SD: standard deviation; NA: not available.

Study	n	Population age	Total yield μg Mean (SD)	Purity A260/A280 Mean (SD)
This study	45	7–15 years old	65.70 (74.49)	2.0 (0.37)
Rogers 2007 [10]	17	24–56 years old	12.6 (7.39)	1.1 (0.07)
Mullot 2005 [11]	20	20–54 years old	3.5 (0.1)	1.6 (0.2)
Saftlas 2004 [18]	15	3–4 months	11.4 (14.2)	1.6 (NA)
Satia-Abouta 2002 [21]	21	≥ 45 years old	11.95 (5.8)	1.8 (NA)
García-Closas 2001 [22]	40	29–74 years old	15.8 (9.8)	1.9 (NA)

**Figure 1:**
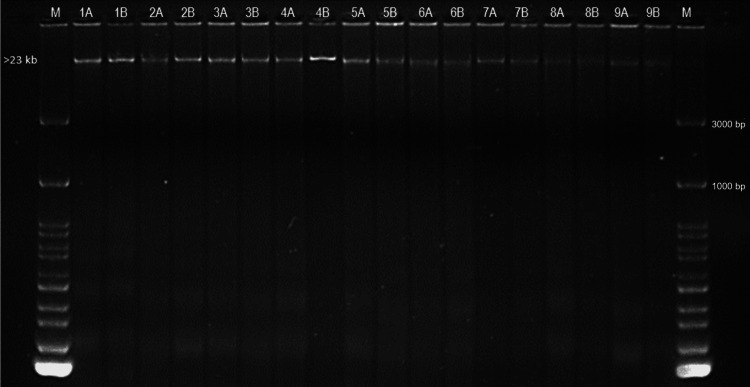
1% electrophoresis gel of genomic DNA from buccal cell DNA obtained by cytobrush. Lane 1 and 20: DNA marker of 100 bp. Immediate extraction: 1A, 2A, 3A, 4A, 5A, 6A, 7A, 8A and 9A. Extraction after eight months of storage at -20°C: 1B, 2B, 3B, 4B, 5B, 6B, 7B, 8B, and 9B.

The integrity of the genomic DNA was determined by ultraviolet transillumination gel electrophoresis visualization. The obtained DNA had a high molecular weight at both processing times. Different band intensities were observed; however, there was no degradation in the DNA that was isolated immediately or in the samples stored for eight months prior to extraction (Figure 1).

**Figure 2:**
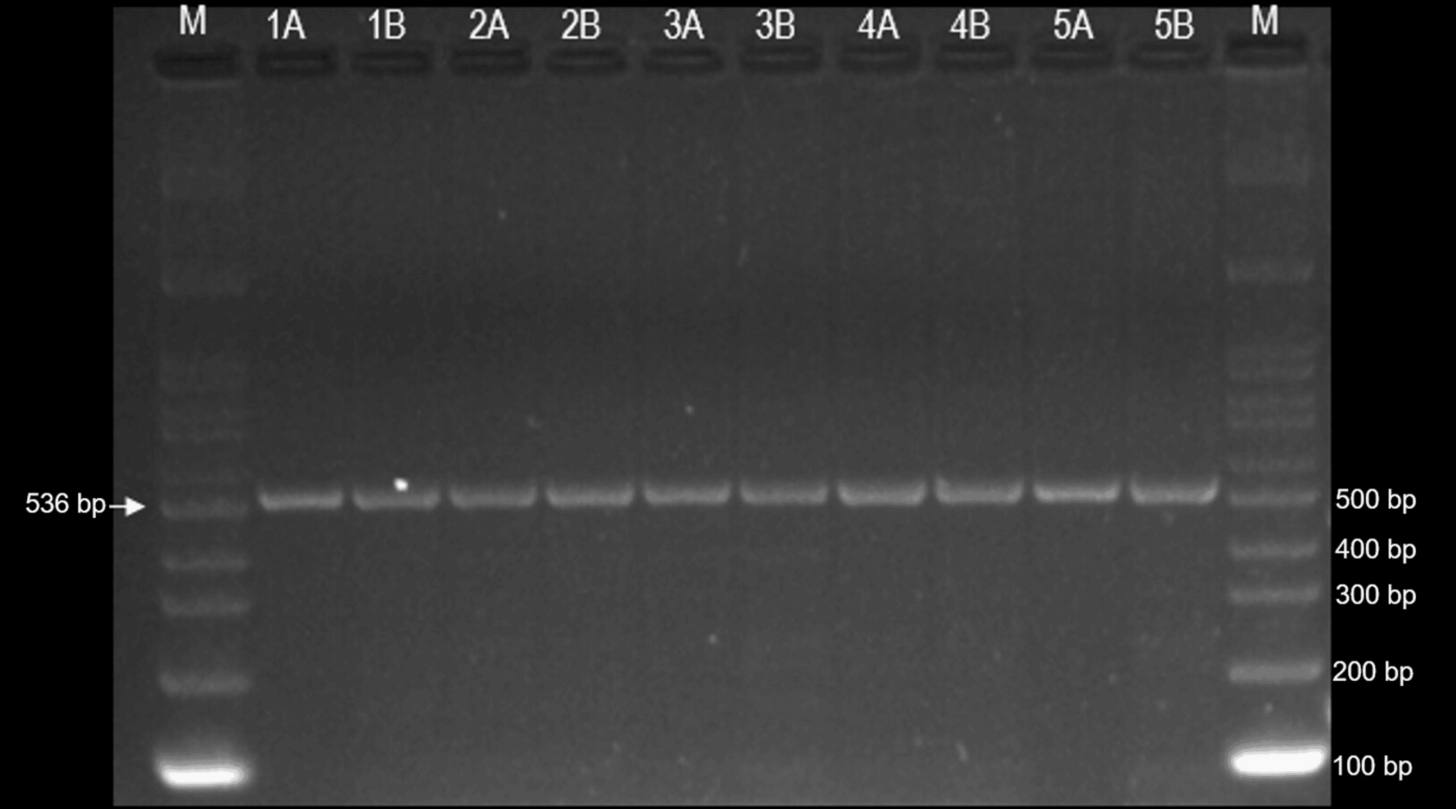
Verification of human β-globin gene PCR products on a 2% agarose gel. Lane 1 and 12: DNA marker of 100 bp. Immediate extraction: 1A, 2A, 3A, 4A and 5A. Extraction after eight months of storage at -20°C: 1B, 2B, 3B, 4B and 5B.

With regard to the amplification of the 536 bp *human β-globin* gene by PCR, the success rates were very high for both samples processed immediately (100%) and those stored for 8 months at -20 °C prior to extraction (97%) (Figure 2).

## Discussion

Different approaches have been developed for the collection and extraction of DNA from buccal cells, as these cells are considered excellent sources of genetic material for molecular analysis [2,3,7-9,11-14,21,22]. However, cytobrush collection could be a noninvasive and cost-effective alternative, especially for large-scale molecular epidemiological studies involving pediatric populations [18,23]. In this study, published methods were modified to develop an optimal protocol for obtaining high-quality DNA from school-aged subjects. Two buccal cell samples were obtained from each subject by cytobrush to determine the effectiveness of the extraction method, as well as when the samples were processed immediately and when they were processed after 8 months of storage at -20 °C.

This is the first publication to date that presents DNA results from cytobrush collections in schoolchildren of these ages (7 to 15 years). Total DNA yields reported in adults from cytobrushes range from 3.5 to 15.8 μg, and the maximum time reported ranges from 6 days to one month [10,11,18,21,24-26]. In comparison, the total yield obtained in this study was greater in both the immediate extraction group (65.70 μg) and the group processed after eight months of storage (47.63 μg). Differences in total DNA yield between the groups were not significant, indicating that although the concentration was lower in the stored samples, a high yield of nucleic acids for analysis was obtained in both groups. This aspect is relevant for large-scale epidemiological studies, and it is not always possible to isolate DNA immediately after field collection. The high yield of nucleic acids is also key in the case of biobanks, as samples must be maintained for certain periods for different assays [4].

The concentration of DNA may depend on the method and time of collection [11]. Other authors used 30 seconds to obtain buccal cells with a cytobrush [20,21]. In the present study, the time for the mucosa to be rubbed with the cytobrush was 15 seconds for each cheek. This action was performed 3 times, for a total of 45 seconds for each left and right side, as reported by Mullot et al. in their study with adults [11]. Although the time considered in our study was longer than that in previous reports, it is noteworthy that this condition was well received by the schoolchildren and allowed sufficient DNA to be collected for analysis. Moreover, the yield obtained using the mouthwash method was also greater than that reported in previous trials involving 12-year-old schoolchildren (17 μg) [13]. Cells that were recovered with rinses or cytobrushes in a shorter time were likely superficial or undergoing apoptosis; therefore, the DNA may be degraded [12].

In contrast to other protocols that kept the samples at room temperature after collection, in this study, the cytobrushes were stored in a cooler and sent to the laboratory to maximize the yield of buccal cells. The group of samples that were not processed on the day of collection were stored at -20°C in the laboratory to preserve integrity, as previously reported by Mullot [11].

According to the literature, the recommended purity of nucleic acid for PCR analysis should be within 1.8 to 2.0 [1]. Consequently, the purity of the isolated DNA was also optimal, at 2.0 and 1.9, respectively, for the extraction times. These values are comparable with those described in previous analyses [21,22,24]. The application of oral hygiene procedures prior to sample collection may contribute substantially to individual differences in nucleic acid purity. Studies that have not included cleaning of the oral cavity prior to collection have reported absorbances of less than 1.8 [10,11,18]. In previous protocols, patients were asked to rinse or brush their teeth thoroughly before sampling or to abstain from food intake [21,23]. In this study, to ensure that food particles were adequately removed, the examiner directly cleaned each subject's mouth with a new toothbrush and pumice stone. Subsequently, the patient was asked to rinse twice with 30 mL of purified water. 

Contaminants and microorganisms during sample collection can have a negative impact on DNA extraction, amplification, and analysis [15,18]. In previous studies, after collection, the cytobrush was immersed in a sterile solution for transport, and until the time of extraction in the laboratory, the brush was resuspended in a lysis buffer, as reported by Mullot and Saftlas in their respective studies [11,18]. From these protocols, purities of less than 1.8 were obtained. In our protocol, the cytobrush was suspended directly in 1 mL of lysis buffer (10 mM Tris-HCl, pH 7.8; 5 mM EDTA; 0.5% SDS) immediately after collection to reduce sample handling and avoid contamination. EDTA in the buffer helps to preserve DNA integrity, as most enzymes involved in nucleic acid degradation require divalent ion cofactors to promote activity [9]. The samples processed after 8 months were immediately frozen at -20°C to avoid bacterial growth.

Researchers have shown that in the DNA extraction process, proteinase K and incubation time are important factors in the recovery of high-quality and high-quantity DNA [27,28]. Authors report using proteinase K for one to three hours to promote protein digestion of cell lysates, removing contamination and releasing nucleic acids in buccal cell samples [8]. However, to enhance the action, we followed Küchler and Aidar in their respective protocols, incubating the samples at 55°C overnight. They obtained quite acceptable yield values, 19.4 and 20.71, respectively [7,9].

The nucleic acid precipitation time was also extended compared to that of other methods, generally ranging from 15 minutes to 2 hours. This step was performed by adding isopropanol and sodium acetate to the samples, which were subsequently stored at -20°C overnight for precipitation; the results corresponded with those of Garcia-Closas, who reported that extended reaction times resulted in better yields [21].

A limitation of this study is that the presence of bacterial DNA was not evaluated. Also, variations in DNA quality that may be due to individual oral flora, dietary habits, or differences in oral mucosal desquamation were not considered. However, when we evaluated the integrity and functional quality of the extracted nucleic acids, our results showed that the DNA is amplifiable for use in genetic studies. Our success in PCR amplification was consistent with the yield and purity obtained. On the other hand, we did not include comparisons with other types of collection methods, such as rinsing or obtaining whole saliva from the same individual, which could be performed in future analyses.

## Conclusions

Through the modifications applied to this method, the results demonstrate its efficacy for obtaining and extracting DNA from buccal cells obtained by cytobrushing. This protocol allows for the obtaining of DNA of high yield, purity, and functional quality for genetic epidemiological studies in schoolchildren, which requires non-invasive and efficient strategies. Furthermore, it was demonstrated that this method can be performed when DNA is extracted immediately after collection and when it is extracted after buccal cell samples are stored for eight months at -20°C.
